# Integrating Indices of Genetic Risk for Cardiovascular Disease∗

**DOI:** 10.1016/j.jacadv.2023.100568

**Published:** 2023-08-24

**Authors:** Michael C. Honigberg, Christian C. Faaborg-Andersen

**Affiliations:** a Cardiology Division, Department of Medicine, Massachusetts General Hospital, Harvard Medical School, Boston, Massachusetts, USA; b Department of Medicine, Massachusetts General Hospital, Harvard Medical School, Boston, Massachusetts, USA; c Program in Medical and Population Genetics, Broad Institute of MIT and Harvard, Cambridge, Massachusetts, USA

**Keywords:** coronary heart disease, familial hypercholesterolemia, genetics, polygenic risk score, risk prediction

**Figure:**
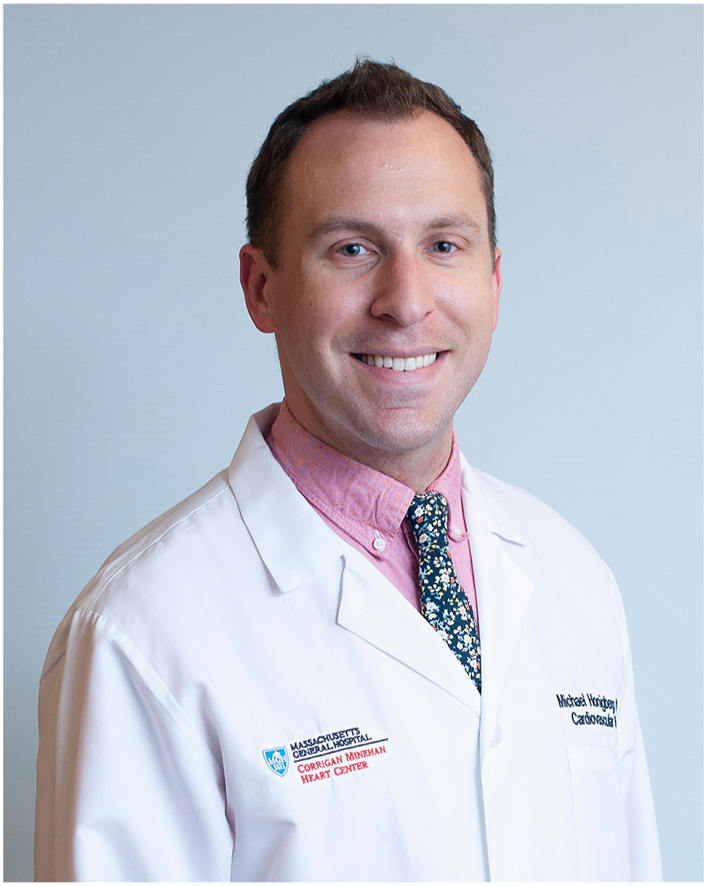

Atherosclerotic cardiovascular disease (ASCVD) is the leading cause of death in the United States and accounts for an estimated 19 million deaths worldwide each year.^1^ Given this significant burden of largely preventable illness, efforts have been made to use cardiovascular risk estimation prior to the development of clinical disease, for example, via the American College of Cardiology/American Heart Association Pooled Cohort Equations (PCEs), to guide allocation of preventive therapies.^2^ While the PCE calculator has proven useful, it remains an imperfect predictor of risk and underperforms among subsets of the population including women^3^ and younger adults.^4^ Indeed, as our understanding of ASCVD risk-enhancing factors has expanded, it has become clear that conventional risk estimation fails to capture certain key predictors of atherosclerosis, including much of the contribution of genetics to heart disease.

In contemporary practice, querying family history of heart disease, especially when premature, represents a commonly used screen for heritable risk, although recent data suggest that the majority of family history of coronary heart disease (CHD) may in fact represent shared nongenetic risk factors, such as environment and group behaviors.^5^ Rare pathogenic variants in key genes involved in cholesterol metabolism, chiefly *LDLR*, *APOB*, and *PCSK9*, underlie classical “monogenic” familial hypercholesterolemia (FH), whereby single gene mutations confer large effects on circulating atherogenic lipoproteins and associated risk of premature ASCVD events. Whether driven by monogenic FH or otherwise, a low-density lipoprotein cholesterol (LDL-C) ≥190 mg/dL represents a Class I guideline indication for statin therapy to reduce lifetime ASCVD risk.^2^ More recently, large genotyped data sets have enabled genome-wide association studies (GWAS) of CHD, implicating biologic pathways that operate independent of conventional ASCVD risk factors. In turn, GWAS have enabled derivation of polygenic risk scores (PRS), which are continuous, normally distributed measures of aggregate genetic risk for disease conferred by variation across the genome (Figure 1). PRS independently predict risk for an array of cardiometabolic diseases,^6^ with the top ∼5% of CHD PRS representing similar risk for CHD as monogenic FH.^7^ Although germline genetic risk is non-modifiable, evidence consistently suggests that addressing other modifiable risk factors is effective at offsetting heightened genetic risk. As elevated PRS is substantially more common than FH, incorporating this genetic information may augment the predictive utility of conventional ASCVD scores.

**Figure 1:**
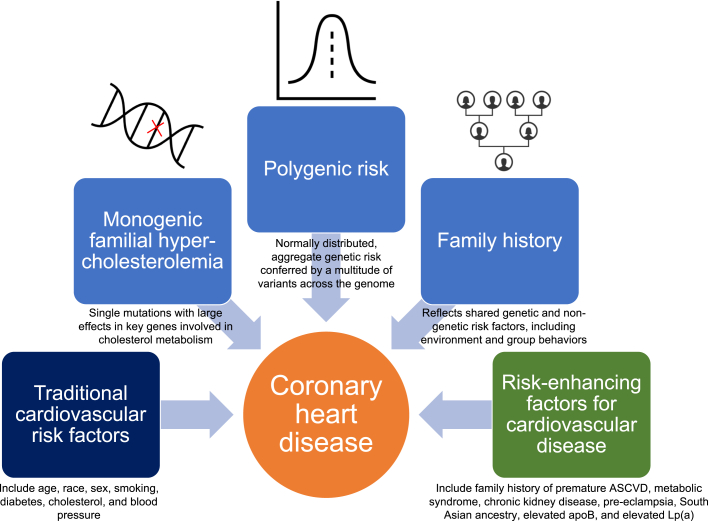
**Polygenic Risk, Monogenic Risk Variants, and Family History Confer Independent Risk for Coronary Heart Disease in the Primary CardiovascularPrevention Population** ASCVD = atherosclerotic cardiovascular disease; Lp(a) = lipoprotein (a).

In this issue of *JACC: Advances*, Saadatagah et al^8^ analyze the utility of CHD PRS in predicting incident CHD, and whether the risks conferred by monogenic FH and history of CHD in a first-degree relative were independent of, and additive to, CHD PRS. In line with current state-of-the-art approaches, the authors used a previously published genome-wide CHD PRS incorporating 1.7 million variants. Among 323,373 primary prevention genotyped individuals in the UK Biobank (UKB), addition of PRS to the PCE improved the C-statistic for CHD from 0.759 to 0.773 (*P* < 0.001), up-classified 5.4% of individuals above the 7.5% threshold of 10-year ASCVD risk, and down-classified 5.7% of individuals below the 7.5% risk threshold. Notably, the up-classified group was younger (56.9 vs 60.8 years) and more likely to be female (60.4% vs 43.8%). Among a subset of approximately 200,000 participants with available whole exome sequences (used to detect FH) in addition to genotype array data (used to calculate PRS), 10,000 individuals had a PRS that fell into the 95th percentile of CHD risk or greater, 673 (0.33%) participants had a pathogenic variant in *LDLR*, *APOB*, or *PCSK9*, and 46,163 (23.0%) carried a first-degree family history of CHD. Risk associated with a PRS ≥95th percentile was independent of FH and family history, and each of the 3 indices of CHD genetic risk conferred additive risk for CHD.

Notably, incorporation of PRS yielded greater improvements in discrimination and net reclassification in younger rather than older adults, a finding that has now been observed across several studies.^9^ This finding implies that incorporation of PRS into risk assessment tools may be particularly useful early in life, whereby high PRS may prompt earlier and more aggressive risk stratification and mitigation, for example, through diagnostic imaging (eg, coronary artery calcium CAC scoring, with PRS-guided CAC scoring currently under study [ACTRN12622000436774]) or more intensive management of modifiable ASCVD risk factors. For example, among young adults (aged 20-39 years) with LDL-C 160-<190 mg/dL, current guidelines endorse using a family history of premature ASCVD to indicate statin eligibility; one might similarly imagine high PRS being used to trigger earlier risk factor modification. Among primary prevention individuals in midlife, family history of premature ASCVD is incorporated as a risk-enhancing factor and CHD PRS may represent an additional risk-enhancing factor to up-classify risk and guide statin prescribing.^10^ Furthermore, among those with established ASCVD (ie, the secondary prevention population), recent data indicate that high PRS may predict residual risk for recurrent ASCVD events,^11^ implying that PRS may signal opportunities to test add-on therapies, treat to lower secondary prevention targets, or enrich clinical trials for events.

Given the promise of genotyping to identify individuals at high risk for ASCVD in advance of clinical disease, what stands in the way of its widespread adoption in practice? Cross-ancestry generalizability of PRS remains a key challenge, as currently available scores are largely trained on genomic data from European ancestry individuals and consequently underperform in other ancestry groups. Similarly, given the demographic composition of the UKB, the present study included primarily European ancestry individuals; as the authors acknowledge, this lack of diversity represents a key limitation, which is common to most studies using the UKB. As PRS become incorporated into clinical practice, underperformance of scores in non-European populations risks perpetuating existing inequities in care.^12^ Increasing the diversity of GWAS used to derive PRS, however, has been shown to substantially improve genetic risk prediction,^13^ including for CHD in a newly published PRS,^14^ and continued efforts toward this end represent a central goal of contemporary genetics research. Additional barriers include cost considerations (although sequencing costs have decreased substantially), logistical challenges, and the need for prospective studies to guide optimal implementation.

Saadatagah et al^8^ are to be commended for their important work demonstrating that polygenic risk, monogenic risk, and family history can each provide additive information about risk in the primary prevention population, and particularly in young adults. These findings add to a growing literature suggesting that genetics may enable more targeted primordial and primary prevention. As ASCVD remains the leading cause of morbidity and mortality, the significance of this potential should not be understated.

## Funding support and author disclosures

Dr Honigberg has received research support from 10.13039/100004328Genentech; consulting fees from CRISPR Therapeutics; advisory board service for Miga Health, all unrelated to this work; and supported by the 10.13039/100000050U.S. National Heart, Lung, and Blood Institute (K08HL166687) and 10.13039/100000968American Heart Association (940166, 979465). Dr Faaborg-Andersen has reported that he has no relationships relevant to the contents of this paper to disclose.
